# Protein engineering by highly parallel screening of computationally designed variants

**DOI:** 10.1126/sciadv.1600692

**Published:** 2016-07-20

**Authors:** Mark G. F. Sun, Moon-Hyeong Seo, Satra Nim, Carles Corbi-Verge, Philip M. Kim

**Affiliations:** 1 Department of Computer Science, University of Toronto, Toronto, Ontario M5S 3E1, Canada.; 2 Terrence Donnelly Centre for Cellular and Biomolecular Research, University of Toronto, Toronto, Ontario M5S 3E1, Canada.; 3 Department of Molecular Genetics, University of Toronto, Toronto, Ontario M5S 3E1, Canada.

**Keywords:** Computational protein design, protein engineering, structural biology

## Abstract

Current combinatorial selection strategies for protein engineering have been successful at generating binders against a range of targets; however, the combinatorial nature of the libraries and their vast undersampling of sequence space inherently limit these methods due to the difficulty in finely controlling protein properties of the engineered region. Meanwhile, great advances in computational protein design that can address these issues have largely been underutilized. We describe an integrated approach that computationally designs thousands of individual protein binders for high-throughput synthesis and selection to engineer high-affinity binders. We show that a computationally designed library enriches for tight-binding variants by many orders of magnitude as compared to conventional randomization strategies. We thus demonstrate the feasibility of our approach in a proof-of-concept study and successfully obtain low-nanomolar binders using in vitro and in vivo selection systems.

## Teaser

Combining the power of combinatorial screening and computational design for optimal protein engineering.

## INTRODUCTION

The success of combinatorial selection strategies is evident from their dominance in the protein engineering field, particularly for the identification of new antibodies against targets associated with cancer and other human diseases. Their success can be attributed to their ability to rapidly construct and screen large libraries to assess protein binding (*1*–*4*). Unfortunately, randomizing more than just eight positions on a protein with all 20 natural amino acids (20^8^ combinations) exceeds the capacity of a typical phage library (≈10^10^), resulting in sequence undersampling that is exponential in the number of randomized positions. Libraries can be biased toward the wild-type sequence or use a reduced set of amino acids because randomly generated sequences are unlikely to yield stable and functional proteins (*1*, *5*, *6*), thus restricting the accessible search space to one that is centered on the wild-type sequence. At the same time, great advances in computational design methodologies have allowed the systematic evaluation of complete sequence landscapes (*7*–*11*). Modern design methods are able to identify novel medium-affinity protein binders (*12*, *13*); however, no current technique efficiently uses computational methods in combination with powerful high-throughput screening systems.

Here, we developed a highly parallel protein engineering approach that, for the first time, directly couples computational protein design with the combinatorial screening capabilities of phage display and yeast two-hybrid (Y2H) using custom microarrays. Custom microarrays have previously been used for transcription factor specificity determination (*14*) and, more recently, in the screening of natural human peptide binders (*15*, *16*). Here, we use microarrays to directly synthesize the computationally designed variants to construct a full-length variant library for high-throughput screening. Our approach contrasts with previous methods whereby computational protein design is used to identify an initial set of variants that likely have the function of interest, such as binding. Experimental strategies are subsequently used to optimize the designed variants, but whose sequence composition is still similar to the designed variants (*17*–*19*). We demonstrate the efficacy of our parallel protein engineering approach by performing positive design on the human ubiquitin–ubiquitin specific peptidase 21 (USP21) complex, by computationally designing 6000 ubiquitin variants for oligonucleotide synthesis and subsequent selection for binding against USP21 by phage display and Y2H. Deep sequencing of the final phage and Y2H pools recovered the variants inhibiting USP21 at low nanomolar concentrations. The approach is highly scalable and generalizable because virtually any protein scaffold can be used in libraries with 100,000s of variants with current technologies.

## RESULTS

### Designing the ubiquitin variant library

**Fig. 1:**
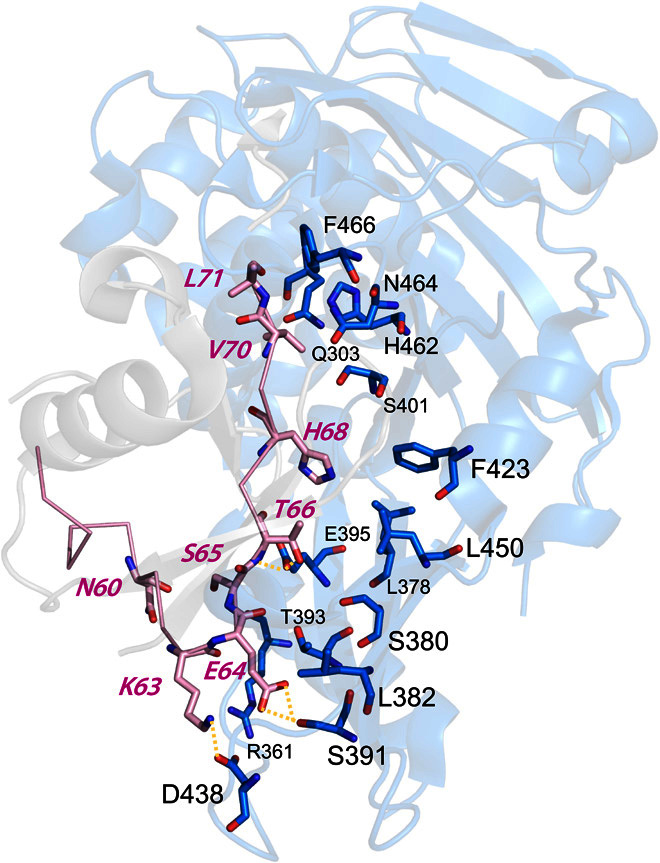
Structure of the ubiquitin-USP21 complex. Ubiquitin and USP21 are shown in gray and blue, respectively, with the engineered ubiquitin region shown in pink.

**Fig. 2:**
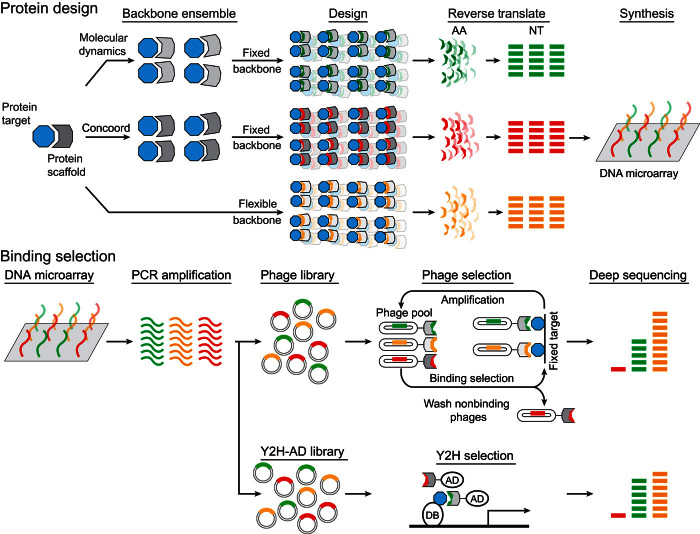
Schematic of the parallel protein engineering strategy. Computational protein design is performed on the human ubiquitin interface (positions 54 to 71) binding USP21 [Protein Data Bank (PDB) ID: 3I3T]. Fixed backbone design is performed on protein ensembles, whereas flexible backbone design is performed directly on the initial crystal structure. Unique sequences (2000), 18 amino acids (AA) in length, were extracted for each of the three design strategies and reverse-translated (NT) for synthesis on a DNA microarray. Libraries were constructed by amplifying the DNA microarray product and were subsequently screened by either phage display or Y2H. Deep sequencing of the final screening products recovered the designed ubiquitin variants that tightly bound USP21.

Using the ubiquitin-USP21 complex as a model, a contiguous 18–amino acid segment on wild-type human ubiquitin (positions 54 to 71) was selected for the design (Fig. 1). Previously, positions 64, 68, and 71 were found to be important for USP21 inhibition; thus, using this system provides us with a set of impartial controls to assess our method (*20*). The complete sequence landscape of the 18 designed positions encompass about 2.6 × 10^23^ possible variants, far exceeding the typical capacities of ≈10^10^ and ≈10^6^ variants for phage display and for Y2H screening libraries, respectively. Rather than creating a library using random sequences biased toward the wild-type sequence, we systematically explore the complete sequence space to select 6000 ubiquitin variants likely to stably fold and bind USP21 by using three computational protein design strategies (Fig. 2).

To capture protein flexibility, we generated alternative protein backbones using two different ensemble generation methods as well as a design strategy that uses flexible backbones. Ensembles of the ubiquitin-USP21 complex were created (i) from a 100-ns molecular dynamic (MD) simulation (*21*) by extracting structures every 40 ps and (ii) from structures whose atoms satisfy multiple atomic distance constraints, such as bond distances, after refining structures whose atoms were randomly initialized within an 8-nm^3^ cube centered on the input positions as applied by the CONCOORD method (*22*). For both the MD and the CONCOORD ensemble methods, 2500 ubiquitin-USP21 complex models were created. To search for optimal sequences for each backbone in the ensemble that may tightly bind USP21, we used the Rosetta fixed backbone design method (*8*), a simulated annealing procedure that samples alternative amino acid side chains whose configurations are specified by the Dunbrack rotamer library (*23*). Finally, we used a flexible backbone approach to identify ubiquitin variants that tightly bind USP21 as employed by the Rosetta backrub method. For this strategy, alternative protein backbones and amino acids are explored within the same sampling scheme, whereby alternative protein backbones are created by rotating atoms around an axis defined by two Cα atoms and alternative amino acid side chains are sampled using the Dunbrack rotamer library (*10*). Starting from the wild-type ubiquitin-USP21 structure [Protein Data Bank (PDB) ID: 3I3T], tight-binding ubiquitin variants are identified using the MD, CONCOORD, and Backrub design strategies. For each design strategy, 62,500 ubiquitin variants were created. The best 2000-scoring designs from each of the three design strategies were selected, for a total of 6000 unique ubiquitin variants tailored for USP21 binding (Fig. 2). These variants were derived from 556, 543, and 2000 backbone models from the MD, CONCOORD, and Backrub protein design strategies, respectively.

**Fig. 3:**
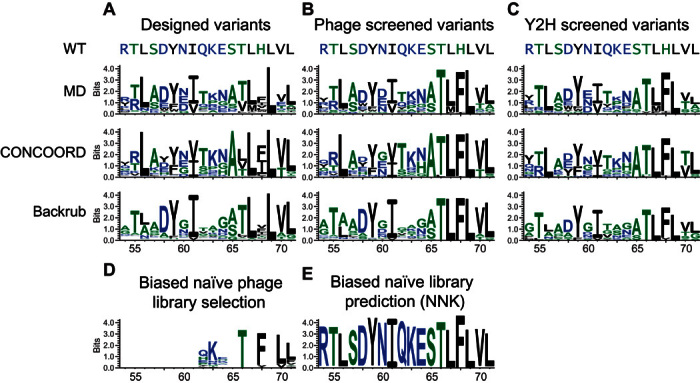
Sequence logos of ubiquitin variants that tightly bind USP21. (**A** to **C**) Sequence logos of computationally designed ubiquitin variants derived from MD, CONCOORD, and Backrub ensembles for (A) 2000 of the best ranked designed variants, (B) variants selected by phage display, and (C) variants selected by Y2H. (**D** and **E**) Sequence logos of ubiquitin variants selected for USP21 binding (D) from a biased naïve library screened by phage display and (E) predicted by random forest models from simulated naïve libraries following the NNK nucleotide randomization scheme. The simulated libraries were biased toward the ubiquitin wild-type nucleic acid composition 70% of the time. The *x* and *y* axes correspond to the designed ubiquitin positions and the information content in bits, respectively, as determined by WebLogo (*39*). Amino acids colored black, green, and blue correspond to hydrophobic, neutral, and hydrophilic residues, respectively.

**Fig. 4:**
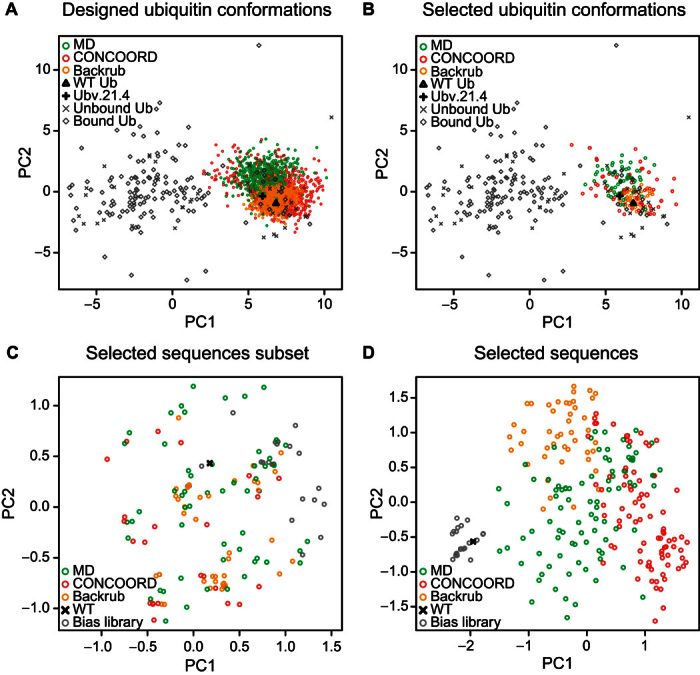
PCA of the designed ubiquitin variants. (**A** and **B**) The first two principal components (PCs), capturing 35% of the variance, are formed from a set of 37 unbound ubiquitin and 137 bound ubiquitin crystal structures, which include the wild-type ubiquitin (WT Ub) and a tight-binding ubiquitin variant (Ubv21.4) bound to USP21. (A) Ubiquitin backbone models from the top 2000 scoring variants from each of the MD, CONCOORD, and Backrub ensembles were projected into the first two PCs. (B) MD, CONCOORD, and Backrub structures associated with the 215 ubiquitin variants found to tightly bind USP21 by phage display are projected into the first two PCs. (**C** and **D**) Sequences of 215 designed variants identified by phage display and 26 variants from a biased naïve library found to tightly bind USP21 in addition to the wild-type ubiquitin sequence were used for PCA over (C) 7 sequence positions (62, 63, 64, 66, 68, 70, and 71) engineered by the biased naïve library (25% of the variance) or (D) all 18 designed positions (21% of the variance).

The MD, CONCOORD, and Backrub designed sequences differed from the wild-type ubiquitin sequence by 53, 58, and 49% (over the 18 designed positions) on average, respectively (Fig. 3A). These designed sequences are actually more similar to variants previously recovered by phage display (*20*) than to the wild-type sequence, as measured by Jenson-Shannon divergence (table S1). Shape complementarity assessment of the ubiquitin backbone models for USP21 binding using principal components analysis (PCA) found that the ubiquitin models are similar to known ubiquitin structures bound to USP21 (PDB ID: 3I3T and 3MTN). However, compared to each other, the designed backbone models occupied different regions in three-dimensional space (Fig. 4A). Furthermore, the median root mean square deviations of the MD, CONCOORD, and Backrub backbones were 0.63, 0.53, and 0.28 Å, respectively, to the wild-type ubiquitin structure (PDB ID: 3I3T) and were 0.64, 0.57, and 0.37 Å, respectively, to Ubv21.4, a known variant that tightly binds USP21 (PDB ID: 3MTN) (*20*). We find that the MD backbones are equally distant from the wild-type and Ubv21.4 ubiquitin structures, whereas the CONCOORD and Backrub backbone models show a bias toward the wild-type structure (fig. S1), which may hinder the identification of tight-binding ubiquitin variants to USP21.

### Parallel screening of the designed ubiquitin library

We constructed a library containing all 6000 computationally designed ubiquitin variants using a custom oligonucleotide array. Specifically, oligonucleotides representing each computationally designed variant were synthesized with a custom microarray and cloned into phage and yeast vectors to create screening libraries (Fig. 2). Phage display and Y2H selection strategies were subsequently performed to assess the binding of the designed ubiquitin variants for tight USP21 binding. In doing so, we combine the targeted search of ubiquitin variants for USP21 binding by computational protein design strategies with the screening capabilities of experimental parallel selection methods.

Tight-binding ubiquitin variants to USP21 were determined by phage display and Y2H selection screens (Fig. 2). For phage display, phage particles selected after four selection rounds had their phagemid polymerase chain reaction (PCR)–amplified and deep-sequenced, recovering 82,292 high-quality sequencing reads, corresponding to 215 unique ubiquitin variants from 88, 82, and 45 variants derived from MD, CONCOORD, and Backrub backbones, respectively (table S3). For Y2H, deep sequencing was performed on PCR-amplified products from the yeast pools surviving on synthetic complete dropout medium [SC-leucine-tryptophan-histidine and 3 mM 3-aminotriazole (3-AT)]. From sequencing, 2280 high-quality sequence reads were recovered, corresponding to 84 unique ubiquitin variants composed of 46, 25, and 13 variants from MD, CONCOORD, and Backrub backbones, respectively (table S3). We observed that the designed variants identified from both phage display and Y2H selection methods had the critical Phe^68^ mutation previously found to be necessary for tight USP21 binding (*20*). Additionally, these selected variants suggest that Thr^66^ also plays an important role in binding. These methods also identified positions 56, 59, 61, 67, and 69 as being important, as indicated by the strong preference for the wild-type amino acid (Fig. 3, B and C). Residues at these positions had their side chains oriented toward the ubiquitin core, giving rise to the observed preference for hydrophobic residues. The phage display and Y2H selection methods identified different ubiquitin variants that tightly bind USP21, with an overlap of 23 to 52% with respect to the Y2H variants (fig. S3). Despite the Y2H library capacity (10^6^) being smaller than those found for a typical phage display library (10^10^), creating a targeted library of computationally designed variants permits alternatives in cell screening strategies to be used, sidestepping the requirement for the laborious expression and purification of the protein targeted by the engineered variants.

### Variant validation

**Fig. 5:**
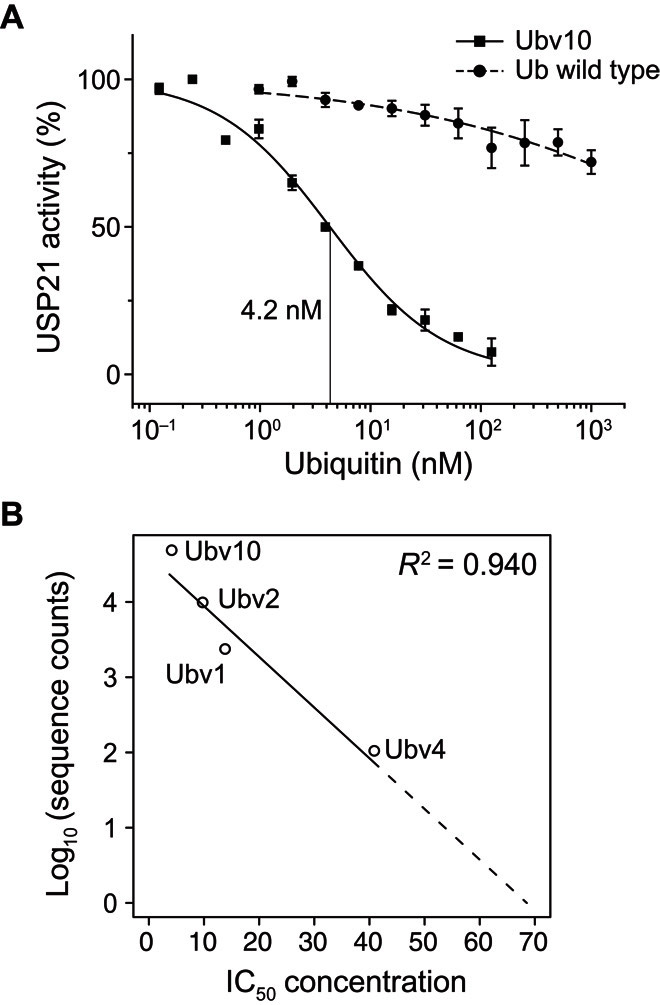
Ubiquitin variant validation. (**A**) Dose-response curve of USP21 activity inhibition by wild-type ubiquitin and the Ubv10 ubiquitin design. IC_50_ concentrations were evaluated as the ubiquitin concentration inhibiting USP21 activity by 50%. The Ubv10 dose-response curve has an IC_50_ value of 4.2 nM. (**B**) A strong correlation exists between the IC_50_ concentrations and the log-transformed deep sequencing counts.

To validate the ubiquitin variants identified by our approach, we first measured the median inhibitory concentration (IC_50_) values (the concentration to inhibit USP21 activity by 50%) of variants recovered after four phage display selection rounds. To this end, 10 infected phage colonies were picked, yielding four unique variants whose geometric IC_50_ mean values were 4.4 nM (Ubv10), 9.9 nM (Ubv2), 13.9 nM (Ubv1), and 40.4 nM (Ubv4) (table S2, Fig. 5A, and fig. S2). The top three binding variants inhibit USP21 1000 times better than the ubiquitin wild type whose IC_50_ value was found to be 18,000 nM (Fig. 5 and table S2). The IC_50_ value of Ubv10 is lower than that of Ubv21.4 (*20*), a USP21 binder generated by conventional phage display, which we found to be 9.4 nM (table S1; its published IC_50_ value is 2.4 nM). To quantify the dissociation constant (*K*_d_) for Ubv10, isothermal titration calorimetry (ITC) was performed, resulting in a *K*_d_ of 42 nM (table S4). Together, these results indicate that our computational design strategy performs similarly or better than conventional experimental strategies, despite validating orders of magnitude of fewer sequences.

**Fig. 6:**
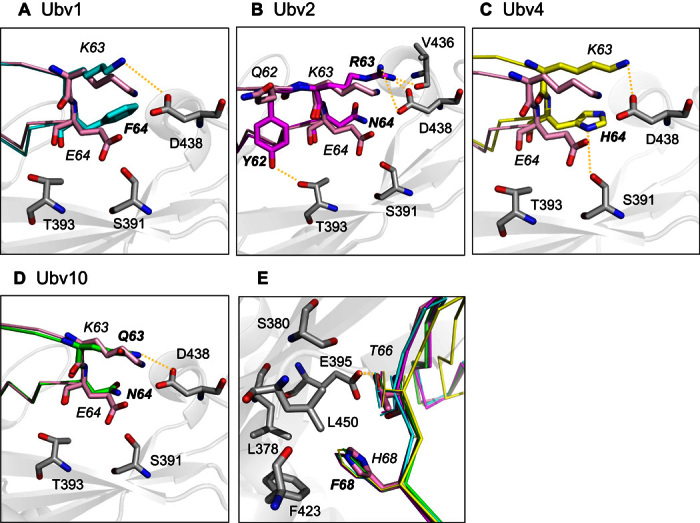
Microenvironment of the ubiquitin-USP21 interface. Molecular models for four ubiquitin variants were superimposed on wild-type ubiquitin (PDB ID: 3I3T). (**A** to **D**) Designed residues (bold) for (A) Ubv1 (cyan), (B) Ubv2 (magenta), (C) Ubv4 (yellow), and (D) Ubv10 (green) are shown relative to wild-type ubiquitin (pink) as colored sticks. USP21 contact residues are shown as gray sticks. Wild-type ubiquitin Glu^64^ was substituted for Phe, Asn, His, and Asn in Ubv1, Ubv2, Ubv4, and Ubv10, respectively, which removes the unfavorable charge interaction with USP21 Asp^438^. Ala^65^ substitution for all variants removes the unfavorable charge interaction with Glu^395^. (**E**) Thr^66^ was maintained in all variants making a hydrogen bond with Glu^395^, whereas Phe^68^ facilitates hydrophobic interactions with USP21 residues Leu^378^, Phe^423^, and Leu^450^.

To rationalize the tight binding of these four variants, we compared their associated structural models by superimposing them onto wild-type ubiquitin that is in complex with USP21 (PDB ID: 3I3T) (Fig. 6). Asp^60^ (Ubv2 and Ubv10), Tyr^62^ (Ubv2), Arg^63^ (Ubv2), and His^64^ (Ubv4) make new hydrogen bonds with USP21, whereas Lys^63^ (Ubv1, Ubv4, and Ubv10) and Thr^66^ (Ubv1, Ubv2, Ubv4, and Ubv10) maintained the original hydrogen bond. Substitution of Glu^64^ (Ub-WT) by Phe (Ubv1), Asn (Ubv2), His (Ubv4), or Asn (Ubv10) contributed to the mitigation of unfavorable charge interactions with Asp^438^ of USP21. Mutating Ser^65^ to Ala^65^ (Ubv1, Ubv2, Ubv4, and Ubv10) abolished close contact with Glu^395^, and Phe^68^ (Ubv1, Ubv2, Ubv4, and Ubv10) enables favorable hydrophobic interaction with side chains of USP21 (Leu^378^, Phe^423^, and Leu^450^).

### Evaluation of the designed sequences

Not all of the 6000 computationally designed ubiquitin variants may be present in the screening libraries; thus, some variants may not have been evaluated for binding against USP21 by phage display or Y2H. To assess all the designed variants, we trained random forest models to predict the variants’ binding affinity for USP21 by using the number of deep sequencing reads as a surrogate measure for binding affinity. First, we observed a strong correlation (Pearson *r* = −0.969; *P* = 0.031) between the log number of deep sequencing reads and the IC_50_ values of the four ubiquitin variants we previously recovered (Fig. 5), rendering the number of deep sequencing reads a useful surrogate measure for a variant’s binding affinity toward USP21. The correlation also suggests, assuming linearity, that after four phage selection rounds, the IC_50_ upper bound for the 215 identified variants is 68.1 nM (Fig. 5). Evaluation of the designed variants was performed using the median of 100 random forest regression models that predicted the log number of deep sequencing reads. The 100 random forest models were trained on the 215 tight-binding variants and a matching number of random sequences that differed for each random forest model. Parameter selection was performed by a grid search over the terminal node size (nodesize) and the number of sampled variables chosen at each decision split (mtry) and was assessed by fivefold cross-validation (see Materials and Methods). The final model identified 92% (24 of 26) of protein variants that tightly bind USP21 from a previous phage display study (*20*), whose sequences were not present among the 215 designed variants used for training. Furthermore, the wild-type and random sequences were predicted to not bind USP21 (fig. S4).

Using the described random forest model, we assessed the quality of our computationally designed library. We found that 16.0% (319 of 2000), 8.5% (169 of 2000), and 5.5% (110 of 2000) of the designed sequences derived from MD, CONCOORD, and Backrub backbones, respectively, are predicted to tightly bind USP21. To ensure that only tight-binding variants were selected, the predicted read count of the Ubv21.4 variant (*20*) was chosen as a threshold (fig. S4). These predicted variants are highly similar to the 215 variants recovered by deep sequencing, indicating that the prediction model captures the necessary sequence signals reflective of low-nanomolar USP21 binding (Fig. 3B and fig. S6). The predicted number of tight-binding variants can also be used to estimate the number of variants that were present in the phage and yeast libraries by taking the ratio between the observed and predicted number of variants. This coarse approximation suggests that 28 to 49% of the designed variants are incorporated within the phage library, whereas 12 to 15% of the designed variants are present in the Y2H library. Note that, for the ubiquitin-USP21 system, protein ensemble design methods outperformed the local backrub sampling strategy. In particular, the fine-grained potential energy function of the AMBER force field used to create the MD protein ensemble generates backbones that are highly amenable for the identification of tight-binding ubiquitin variants for USP21, despite the protein ensemble being derived from the wild-type sequence.

Next, we sought to understand the limitations imposed by naïve (that is, combinatorial) libraries used by conventional engineering methods, such as phage display and other selection strategies. To this end, we generated 10 sets of 1 million random sequences following the NNK randomization (N ∈ {A, C, G, T} and K ∈ {G, T}) strategy, while sampling the wild-type nucleotide 70% of the time, as similarly used for the creation of large biased libraries (*5*, *20*). Evaluating all the randomized sequences using the previously described random forest model for tight binding (Fig. 3E), we found that the MD, CONCOORD, and Backrub methods enrich for the number of tight-binding variants by 14906.5, 7897.2, and 5140.2. That is, a computationally generated library of 90,000 variants (easily accessible using modern oligonucleotide synthesis strategies) would yield as many or more good binders as a standard random phage library of around 10^9^ variants while also offering many advantages, such as full control over the biophysical properties as well as enabling of in-cell screening.

The large number of validated ubiquitin variants enables us to quantitatively evaluate structural features necessary for tight binding. Unfortunately, ranking computationally designed variants with respect to binding affinity is a difficult problem because of (i) approximations made within scoring functions and (ii) the fact that structures used for design are static instances of a dynamic protein. Hence, not surprisingly, no strong correlation exists between the deep sequencing read counts and the Rosetta energy scores of the ubiquitin variants associated with MD, CONCOORD, and Backrub backbones. A similar observation is found when comparing the Δ Rosetta energy scores of the variant and wild-type structures. Difficulties associated with designing protein-protein interaction interfaces to enhance interaction affinity (*24*, *25*) prompted the development of scoring functions specific for protein interface design, whereby energy terms of existing functions were reweighted or a reduced set of terms focused on electrostatics and solvation were used (*26*–*28*). Alternatively, increasing the score contribution of residues within the interface was found to enhance the recovery of experimentally determined variants (*29*). To this end, we searched over different combinations of energy terms within the Rosetta scoring function and positions on the protein interface. No correlation was found when simply considering residues within 5 Å of the designed residues on the ubiquitin interface. However, focusing on residue side chains on the ubiquitin interface (positions 60, 63, 64, 65, 66, 68, 70, and 71) oriented towards the USP21 interface that are within 5 Å of the designed ubiquitin positions, can a significant correlation be found. By only considering the Lennard-Jones attractive and repulsion terms from the Rosetta energy function one finds a strong correlation between the deep sequencing read counts and energy scores, as observed from the Spearman correlation of −0.30 (*P* = 0.0041), −0.33 (*P* = 0.0024), and −0.30 (*P* = 0.045) for MD, CONCOORD, and Backrub designs, respectively. Indicating that after a design procedure a reranking step using only a Lennard-Jones potential may be beneficial when attempting to maximize the number of protein variants that tightly bind a protein target.

## DISCUSSION

We have described a general parallel protein engineering strategy by integrating high-throughput computational protein design approaches, oligonucleotide synthesis, parallel screening methods, deep sequencing, and subsequent computational analysis. In doing so, we combined the targeted search of ubiquitin variants for USP21 binding by computational protein design methods with the screening capabilities of experimental parallel selection methods. By directly constructing large screening libraries composed of full-length designed variants, our strategy enables us to sidestep the difficult issues of (i) ranking variants with a computationally expensive scoring function and (ii) testing only a few highly ranked variants.

We demonstrated our parallel protein engineering approach by experimentally screening 6000 computationally designed ubiquitin variants predicted to bind USP21. We find that all attempted computational design strategies (MD, CONCOORD, and Backrub) successfully identified multiple ubiquitin variants that tightly bind USP21. These tight-binding variants occupy distinct regions in sequence space depending on the computational design strategy (Fig. 4, C and D, and fig. S4, A and B), suggesting that multiple protein design strategies are necessary to fully explore the sequence landscape because each design method has distinct biases. Because the same search and scoring functions were used to identify tight binders from the MD and CONCOORD protein ensembles, the observed performance differences among the protein ensemble design strategies can be attributed to the different protein backbone conformations being explored by each ensemble generation method (Fig. 4B). We have shown that protein ensembles initialized with the wild-type structure can be used to identify many tight-binding variants. Whereas the backrub flexible backbone design approach generated ubiquitin conformations highly similar to the wild-type structure (Fig. 4, A and B), the recovered variants had a sequence composition distinctly different from the wild-type sequence (Fig. 3, A to C). This limited exploration of alternative protein backbones is due to the short backrub trajectories used in this study because only structures similar to the wild-type structure are explored (Fig. 4A). Furthermore, exploration of both backbone and sequence space in a single trajectory may hinder the identification of tight-binding variants because low temperatures may be necessary for the recovery of tight-binding variants, whereas higher temperatures may be required for the rapid exploration of alternative protein backbones.

We find that creating a targeted library by computational design methods enables diverse sequences to be evaluated that have tight binding to USP21 as assessed by phage display and Y2H selection experiments (Figs. 3 and 4, C and D). On the other hand, the naïve library had a limited diversity because of the wild-type sequence being sampled 70% of the time at each nucleotide position. Although biasing for sequences that resemble the wild-type increases the number of variants likely to form a folded protein, it restricts the accessible sequence space that a screening strategy can search. The large variation in sequence composition of the variants found to tightly bind USP21 suggests that the ubiquitin sequence landscape capable of low-nanomolar affinity for USP21 is large and not at all confined to sequences similar to those of the wild type (Fig. 3, B and C).

Our predictions illustrate that current scoring functions and search strategies used in computational protein design are efficient in searching the complete sequence space and honing in on regions containing nanomolar protein binders but continue to have difficulty identifying very high affinity binders. That is, current scoring functions can distinguish “good” from “bad” binders but are still poor at distinguishing “very good” from “good.” Our methodology rectifies the problem by testing thousands of variants.

Computational design methods have been shown to successfully work in a broad range of systems (*12*, *13*, *30*), indicating that our parallel engineering strategy will be broadly applicable. However, because our strategy is reliant on computational methods to create the screening library, its inherent limitation is the availability of suitable protein structures. This restriction can be partially overcome with homology modeling and other prediction methods. As additional structures are deposited into the PDB, our parallel engineering strategy will become more applicable to a greater number of protein systems. The use of a random forest model to identify variants not discovered by the experimental screening methods but are likely to tightly bind USP21 may have limited success because of (i) the number of positions responsible for tight binding and (ii) the number of tight-binding variants recovered by the screening methods that can be used as training.

Recovery of ubiquitin variants inhibiting USP21 with low nanomolar IC_50_ concentrations validates our strategy and illustrates that a small 6000 computationally designed library can recover hundreds of variants whose sequences are diverse, yet tightly bind a targeted protein. The ability to rapidly ascertain computationally designed proteins for binding will be invaluable to the design community as they continue to refine their methodologies and scoring functions for variant ranking. With current microarray synthesis technologies, our approach can design, synthesize, screen, and recover 90,000 protein variants at a reasonable cost. As microarray synthesis technology advances, both the number of variants and oligonucleotide length will continue to increase, enabling larger protein regions to be explored in ever larger libraries. Coupled with the increase in computational power and the continued development of new protein design and deep sequencing strategies, our parallel protein engineering approach will have a broad impact to the development of targeted protein binders in both diagnostic and therapeutic settings.

## MATERIALS AND METHODS

### Model preparation

The human wild-type ubiquitin–USP21 complex (PDB ID: 3I3T) was used as the initial structure for both ensemble and flexible computational protein design strategies (*20*). Unresolved loop segments in the USP21 protein structure (residues 209 to 564) were completed with the FALC-Loop web server (*31*). To ensure that asparagine, glutamine, and histidine amino acids were correctly oriented, any position with strong evidence for flipping was corrected by MolProbity4 (*32*).

Protein backbone ensembles were generated from a 100-ns MD simulation and from distant constraints using CONCOORD (*22*). In total, 2500 backbones were generated for each ensemble. An explicit solvent MD simulation was run with the GROMACS package (*21*) using the AMBER force field (*33*) and the TIP3P water model. Fifty-one Na^+^ ions and 61 Cl^−^ ions were added to the solution to achieve a neutrally charged system. The ubiquitin-USP21 complex was minimized using the steep integrator for 5000 steps, with particle mesh Ewald (PME) electrostatics using 1.2 Å cutoffs. The system was equilibrated at constant volume/temperature then at constant pressure/temperature (Parrinello-Rahman/V-rescale couplings) at 300 K for 5 ps each. The ensemble used for fixed backbone design is composed of structures sampled every 40 ps over the 100-ns trajectory. The ubiquitin-USP21 complex used as input for CONCOORD had its hydrogen atoms removed and replaced with the reduce program from MolProbity4 (*32*). The CONCOORD dist command was run with parameters “-r -m 100,” and the ensemble was subsequently generated with the disco command using parameters “-bump -dyn 1.”

To prepare the ensembles for fixed backbone design and the initial structure for flexible design to be used with the Rosetta energy function, each chain was separately minimized with the Rosetta relax program with the flags “-relax:constrain_relax_to_start_coords -relax:coord_constrain_sidechains -relax:ramp_constraints false -ex1 -ex2 -use_input_sc -flip_HNQ -no_optH false -dun10 true -score:weights talaris2013.” The ubiquitin-USP21 complex was reconstructed by merging the two minimized protein structures. Here, we used the Rosetta software suite version 3.5 (build 2013 week 42) with the Dunbrack 2010 rotamer library (*23*).

### Computational protein design of the ubiquitin interface

Ubiquitin positions 54 to 71 were selected for computational protein design and could be varied in any of the 20 canonical amino acids. Residues within a 10 Å distance from ubiquitin residues 54 to 71 were allowed to alter their residue rotamers. The remaining residues had their rotamers fixed during the design process, thereby limiting memory consumption. Fixed backbone design was run 25 times for each minimized structure in the ensemble, resulting in 62,500 designed protein models, using the flags “-ex1 -ex2 -dun10 true -score:weights talaris2013 -no_his_his_pairE -extrachi_cutoff 0 -multi_cool_annealer 10 -nstruct 25.” For flexible backbone protein design, Rosetta backrub was executed with the flags “-ex1 -ex2 -dun10 true -score:weights talaris2013 -extrachi_cutoff 0 -no_his_his_pairE -backrub:ntrials 10000 -nstruct 25,” resulting in 62,500 designed protein models. For both ensemble and flexible protein design strategies, the resulting designs underwent a further round of minimization with the same relaxation flags as above, but the complete complex was minimized. Computations were performed on the GPC supercomputer provided by the SciNet HPC Consortium (*34*).

### Oligonucleotide synthesis

Oligonucleotides corresponding to the 6000 computational designed variants were synthesized using a custom microarray ordered from CustomArray.

### Phage display screening

The ubiquitin variant library was constructed using oligonucleotide-directed mutagenesis (*35*). The oligonucleotide library (0.6 μg) was 5′-phosphorylated for 1 hour at 37°C in TM buffer [10 mM MgCl_2_, 50 mM tris-HCl (pH 7.5)] with 1 mM adenosine 5′-triphosphate (ATP), 5 mM dithiothreitol (DTT), using a T4 polynucleotide kinase (1 U/μl; New England Biolabs). The phosphorylated oligonucleotides were annealed to single-strand template DNA (20 μg) containing the wild-type ubiquitin sequence fused with the M13 major coat protein P3 [described by McLaughlin and Sidhu (*36*)] by incubating at 90°C for 3 min, at 50°C for 3 min, and at 20°C for 5 min. Complementary DNA primed by the phospho-oligonucleotides was synthesized and ligated by the addition of 10 μl of 10 mM ATP, 10 μl of a 10 mM deoxynucleotide triphosphate mixture, 15 μl of 100 mM DTT, 30 Weiss units of T4 DNA ligase, and 30 U of T7 DNA at 20°C overnight. The double-stranded DNA was purified using a QIAquick DNA purification kit and transformed into *Escherichia coli* SS320 cells preinfected with M13KO7 helper phage. The cells were grown overnight in 500 ml of 2YT medium at 37°C. The supernatant was precipitated by the addition of one-fifth volume of polyethylene glycol (PEG)–NaCl (20% PEG-8000 and 2.5 M NaCl) and incubated for 5 min at 4°C and spun down at 28,880*g* at 4°C for 20 min. The phage pellet was resuspended with 20 ml of PBT [phosphate-buffered saline (PBS), 0.05% Tween 20, and 0.2% bovine serum albumin (BSA)] and stored at −80°C with 20% glycerol.

Phage selection was performed as described elsewhere (*15*) with minor modifications. Four wells of 96-well Maxisorp immunoplates were coated with 100 μl of neutravidin (5 μg/ml) overnight at 4°C and blocked with 200 μl of blocking buffer (PBS and 0.2% BSA) for 1 hour at 4°C, followed by incubation with 100 μl of biotinylated USP21 (10 μg/ml) (*20*) for 30 min at room temperature (*20*). Ubiquitin phage library (100 μl) was added to the coated plate and incubated for 1 hour at room temperature, with gentle shaking. After washing four times with PT buffer (PBS and 0.5% Tween 20), bound phages were eluted by adding 100 μl of 100 mM HCl to each well, followed by 5-min incubation. Eluted phages were neutralized with 1:10 volume of 1 M tris-HCl (pH 11), and 2 ml of *E. coli* OmniMax in log phase (*A*_600_ = ~0.8) was then infected with 200 μl of the eluted phages for 30 min by incubation at 37°C with shaking at 200 rpm. A final concentration of 10^10^ phage per microliter of M13KO7 helper phage was added to the culture and incubated for 45 min at 37°C with shaking at 200 rpm. The culture was transferred to 50 ml of 2YT with kanamycin (25 μg/ml) and carbenicillin (100 μg/ml) and incubated overnight at 37°C and shaken at 200 rpm. The cultivated cells were pelleted by spinning at 17,090*g* for 10 min, and phage particles were precipitated with PEG-NaCl for the following selection. Ninety-six infected colonies were randomly picked from both selection rounds 3 and 4 and grown in 96-well deep well plates (containing 400 μl of 2YT medium with carbenicillin and M13KO7) overnight at 37°C with shaking at 200 rpm. One hundred ninety-two wells of 384-well Maxisorp immunoplate were coated with 30 μl of neutravidin (10 μg/ml), and another 96 wells were coated with BSA (5 μg/ml) overnight at 4°C. After blocking the plates with 60 μl of blocking buffer for 1 hour, 96 wells coated with neutravidin were incubated with 30 μl of biotinylated USP21 (10 μg/ml) for 30 min at room temperature. The phage supernatant of 96 colonies was subsequently transferred to fresh tubes and diluted threefold with PBT buffer, followed by incubation onto the protein-coated 384-well plates for 1 hour at room temperature with gentle shaking. After washing eight times with PT buffer, 30 μl of horseradish peroxidase (HRP)/anti-M13 antibody conjugate (1:5000 dilution in PBT) was added to each well and then incubated for 30 min at room temperature with gentle shaking. Trimethylboron substrate (30 μl) was added to the washed wells, and color development was monitored for 15 min. HRP reaction was quenched by adding 30 μl of 1.0 M H_3_PO_4_, and the plates were spectrophotometrically read at 450 nm using a plate reader. Clones showing four times higher enzyme-linked immunosorbent assay signal of USP21 than neutravidin and BSA were used for Sanger sequencing.

### Y2H screening

USP21 and ubiquitin phage library were transferred by Gateway cloning (Invitrogen) into Y2H destination vectors pDEST-DB and pDEST-AB, respectively. Transformation of DB-USP21 and AD-Ubiquitin library constructs into *Saccharomyces cerevisiae* was performed using the LiAc/SS Carrier DNA/PEG method (*37*). Cells (2.5 × 10^8^) from overnight culture of Y8800 (MATa) and Y8930 (MATα) were added to 50 ml of the prewarmed 2× YPAD medium (final, 5 × 10^6^ cells/ml). Cells were cultivated at 30°C until the cell titer is 2 × 10^7^ cells/ml. Cells were spun down at 3000*g* for 5 min and washed two times with 25 ml of sterile water. Cell pellets were resuspended with 1 ml of sterile water and were pelleted again by centrifugation. Cells were finally resuspended with 0.5 ml of sterile water and mixed with transformation mix [240 μl of 50% PEG-3500, 36 μl of 1.0 M LiAc, 50 μl of SS carrier DNA (2 mg/ml), and 34 μl of plasmid DNA/water mixture]. DB-X or AD-Y constructs were transformed into Y8930 or Y8800, respectively. After vigorous vortexing, the resuspended mixture was incubated for 40 min at 42°C. Pelleted cells were resuspended with 1 ml of sterile water and plated onto selection plates (-Leu or -Trp synthetic dropout media). To make diploid cells for Y2H screening, the same number of cultured cells of each haploid was mixed and cultivated on 2× YPAD plates. Final diploid cells (DB-USP21 + AD-Ubiquitin library/DB-USP21 + AD-null) were tested on four different synthetic complete dropout media with various concentrations of 3-AT (-Leu-Trp/-Leu-Trp-His/-Leu-Trp-His + 1 mM 3-AT/-Leu-Trp-His + 3 mM 3-AT). After 4 to 6 days of incubation at 30°C, fast-growing colonies from 3 mM 3-AT plates were picked and regrown on -Leu-Trp plates for Sanger sequencing, whereas all cells on selection plate were scrapped and used as a template for deep sequencing. Picked colonies were lysed by using zymolyase solution for 3 hours at 37°C followed by 15 min at 95°C and were then PCR-amplified using a pair of primers for Sanger sequencing. Selected yeast pools were used to purify plasmid pools using Zymoprep II (Zymo Research).

### Deep sequencing

The phage pools of rounds 3 and 4, the naïve phage library, and pooled plasmid from Y2H were barcoded for Illumina sequencing as previously described (*15*). Briefly, phage pools (5 μl) were used as templates for 24 cycles of 50-μl of PCRs using barcoded primers for each reaction (0.5 μM for each forward and reverse) and KAPA HiFi HotStart ReadyMix (Kapa Biosystems). The PCR product were confirmed by gel electrophoresis (2% agarose gel) by loading 1 μl of the sample with 6× loading buffer, and the concentration of each PCR product was quantitated using Quant-iT PicoGreen assay (Invitrogen) (*15*). PCR products were pooled as equal quantities and purified using a QIAquick gel extraction kit. Pooled amplicons were quantified using Quant-iT PicoGreen assay (Invitrogen). The insert size of the pooled library was confirmed on an Agilent Bioanalyzer High Sensitivity DNA chip (Agilent Technologies), and the size-corrected concentration was determined with reverse transcription quantitative PCR (Kapa Biosystems Illumina standards). Libraries (11.4 pM) and the PhiX control library (0.6 pM) (Illumina) were denatured and loaded on a MiSeq V2 sequencing kit, with a paired-end read length of 250 base pairs.

High-quality deep sequencing reads of the phage selection pools and Y2H pooled plasmids were identified by selecting short reads whose minimum Phred score was greater than 30. For the Y2H selection, three positions were permitted to have a minimum Phred score of 20. To mitigate the number of false positives, ubiquitin variants associated with greater than or equal to 2 and 5 reads counts were used for the phage selection pools and Y2H pooled plasmids, respectively.

### Purification of protein (USP21 and Ubv)

Selected ubiquitin variants and USP21 were expressed in *E. coli* BL21(DE3) and were cultivated to express proteins. Protein expression was induced by 0.5 mM isopropyl-β-d-thiogalactopyranoside at mid-log phase. After growing the culture overnight at 16°C, the cells were harvested by centrifugation at 14,000*g* for 10 min. The cells were lysed with a sonicator, and proteins were purified using Ni–nitrilotriacetic acid agarose (Qiagen) according to the product manual. Concentration of the purified proteins was determined by measuring the absorption at 280 nm.

### USP21 inhibition assay (IC_50_)

USP21 enzymatic assay was performed with the C-terminal derivatization of ubiquitin with 7-amido-4-methylcoumarin (Ub-AMC) (Boston Biochem). Inhibitory effects of the ubiquitin variants were measured in the assay buffer [50 mM Hepes (pH 7.5), 0.01% Tween 20, and 10 mM DTT] with 1 μM Ub-AMC, 15 nM USP21, and varying concentrations of the ubiquitin variants. USP21 and ubiquitin variants were mixed in the assay buffer and incubated for 30 min at room temperature. The reaction was started by the addition of Ub-AMC. Proteolytic activity of USP21 was observed by the increase of fluorescence emission at 460 nm with excitation at 360 nm for 30 min using a BioTek Synergy 2 plate reader (BioTek Instruments). Activity of USP21 was normalized to noninhibited USP21, and the percent activity was plotted versus the ubiquitin variant concentrations with a log scale. IC_50_ values were calculated by fitting a dose-response curve with a four-parameter logistic function.

### Isothermal titration calorimetry

The purified proteins were dialyzed overnight at 4°C against 50 mM Hepes buffer (pH 7.0) with 500 mM NaCl and 1 mM 2-mercaptoethanol. Calorimetric titrations were carried out using a MicroCal ITC_200_ (Malvern), with an operating cell volume of 205.9 μl. Three independent titrations were performed using the same protein batch with a concentration of 25 μM USP21 in the cell and 339.2 μM for Ubv10. Protein concentration was determined by absorbance at 280 nm using an extinction coefficient of 32,890 M^−1^ cm^−1^ and 7450 M^−1^ cm^−1^ for USP21 and Ubv10, respectively. The syringe was stirred at a speed of 600 rpm to ensure rapid mixing in the cell. Experiments were carried out at 25°C. Each titration was initiated with a 0.2-μl injection, followed by 25 to 40 injections spaced 150 s, of 1 to 2 μl. The binding stoichiometry (*N*), association constants (*K*_a_), and binding enthalpy (Δ*H*) were obtained by nonlinear regression analysis using a one-independent-type-of-sites binding model implemented in the Origin 7.0 software.

### Predicting deep sequencing read counts from sequence

The random forest regression models were built using the R (version 2.14.1) randomForest package (*38*). Positive training data were formed from the 215 sequences recovered from deep sequencing the phage pool after four selection rounds. An equal number of negative training samples were randomly generated using all 20 natural amino acids over the 18 designed positions and assigned a sequence read count of 1 × 10^−10^. Parameters were selected using a grid search and fivefold cross-validation over the terminal node size, and the number of sampled variables chosen at each decision split was selected to be 3 and 7, respectively. Here, an equal number of tight-binding variants and random sequences, 45 samples each (90 total), were chosen for each fold. This validation approach was chosen to ensure that enough testing data were available for each fold when using the Spearman rank correlation as an evaluation metric (fig. S5). In total, 100 random forest models were trained. This ensemble of random forests is used to predict the deep sequencing counts for all 6000 designed sequences. A count threshold of 0.6125 is used as a lower bound to determine the percentage of the designed sequences that inhibit USP21 below the IC_50_ concentration of 68.1 nM.

## Supplementary Material

http://advances.sciencemag.org/cgi/content/full/2/7/e1600692/DC1

## SUPPLEMENTARY MATERIALS

Supplementary material for this article is available at http://advances.sciencemag.org/cgi/content/full/2/7/e1600692/DC1 fig. S1. Structural comparisons of the ubiquitin backbone models. fig. S2. IC_50_ and affinity validation of a subset of the designed ubiquitin variants against USP21. fig. S3. Venn diagrams of the designed ubiquitin variants recovered by phage display and Y2H. fig. S4. PCA of sequences identified by Y2H. fig. S5. Random forest regression model for sequence count prediction. fig. S6. Sequence logos of ubiquitin variants predicted to tightly bind USP21 by an ensemble of random forests model for variants derived from MD, CONCOORD, and Backrub. fig. S7. Y2H screening of ubiquitin library against USP21. table S1. Jenson-Shannon divergence of designed ubiquitin variants derived from MD, CONCOORD, and Backrub ensembles compared to the wild-type sequence and ubiquitin variants recovered from a biased naïve library. table S2. IC_50_ and associated deep sequencing read counts for four selected low-nanomolar binders to USP21. table S3. Deep sequencing read counts of ubiquitin variants surviving phage display and Y2H selections. table S4. Isothermal titration calorimetry of Ubv10 binding USP21.
