# Supplementary material for: Protein engineering by highly parallel screening of computationally designed variants
Source: Sci Adv. 2016 Jul 20;2(7):e1600692. doi: 10.1126/sciadv.1600692 (PMC4956399; doi:10.1126/sciadv.1600692)
Supplement: http://advances.sciencemag.org/cgi/content/full/2/7/e1600692/DC1 [file supp_2_7_e1600692__index.html]

Science Advances | Science Advances

## Supplementary Materials

**This PDF file includes:**

- fig. S1. Structural comparisons of the ubiquitin backbone models.
- fig. S2. IC50 and affinity validation of a subset of the designed ubiquitin variants against USP21.
- fig. S3. Venn diagrams of the designed ubiquitin variants recovered by phage display and Y2H.
- fig. S4. PCA of sequences identified by Y2H.
- fig. S5. Random forest regression model for sequence count prediction.
- fig. S6. Sequence logos of ubiquitin variants predicted to tightly bind USP21 by an ensemble of random forests model for variants derived from MD, CONCOORD, and Backrub.
- fig. S7. Y2H screening of ubiquitin library against USP21.
- table S1. Jenson-Shannon divergence of designed ubiquitin variants derived from MD, CONCOORD, and Backrub ensembles compared to the wild-type sequence and ubiquitin variants recovered from a biased naïve library.
- table S2. IC50 and associated deep sequencing read counts for four selected low-nanomolar binders to USP21.
- Legend for table S3
- table S4. Isothermal titration calorimetry of Ubv10 binding USP21.

Download PDF

**Other Supplementary Material for this manuscript includes the following:**

- table S3 (Microsoft Excel format). Deep sequencing read counts of ubiquitin variants surviving phage display and Y2H selections.

**Files in this Data Supplement:**

- Adobe PDF - 1600692\_SM.pdf
