# Supplementary material for: Protein engineering by highly parallel screening of computationally designed variants
Source: Sci Adv. 2016 Jul 20;2(7):e1600692. doi: 10.1126/sciadv.1600692 (PMC4956399; doi:10.1126/sciadv.1600692)
Supplement: http://advances.sciencemag.org/cgi/content/full/2/7/e1600692/DC1 [file 1600692_SM.pdf]

Science Advances

AAAS

advances.sciencemag.org/cgi/content/full/2/7/e1600692/DC1

Supplementary Materials for

Protein engineering by highly parallel screening of computationally designed variants

Mark G. F. Sun, Moon-Hyeong Seo, Satra Nim, Carles Corbi-Verge, Philip M. Kim

Published 20 July 2016, *Sci. Adv.* **2**, e1600692 (2016)
DOI: 10.1126/sciadv.1600089

The PDF file includes:

- fig. S1. Structural comparisons of the ubiquitin backbone models.
- fig. S2. IC_{50} and affinity validation of a subset of the designed ubiquitin variants against USP21.
- fig. S3. Venn diagrams of the designed ubiquitin variants recovered by phage display and Y2H.
- fig. S4. PCA of sequences identified by Y2H.
- fig. S5. Random forest regression model for sequence count prediction.
- fig. S6. Sequence logos of ubiquitin variants predicted to tightly bind USP21 by an ensemble of random forests model for variants derived from MD, CONCOORD, and Backrub.
- fig. S7. Y2H screening of ubiquitin library against USP21.
- table S1. Jenson-Shannon divergence of designed ubiquitin variants derived from MD, CONCOORD, and Backrub ensembles compared to the wild-type sequence and ubiquitin variants recovered from a biased naïve library.
- table S2. IC_{50} and associated deep sequencing read counts for four selected low-nanomolar binders to USP21.
- Legend for table S3
- table S4. Isothermal titration calorimetry of Ubv10 binding USP21.

Other Supplementary Material for this manuscript includes the following:
(available at advances.sciencemag.org/cgi/content/full/2/7/e1600692/DC1)

- table S3 (Microsoft Excel format). Deep sequencing read counts of ubiquitin variants surviving phage display and Y2H selections.

Supplementary Figures

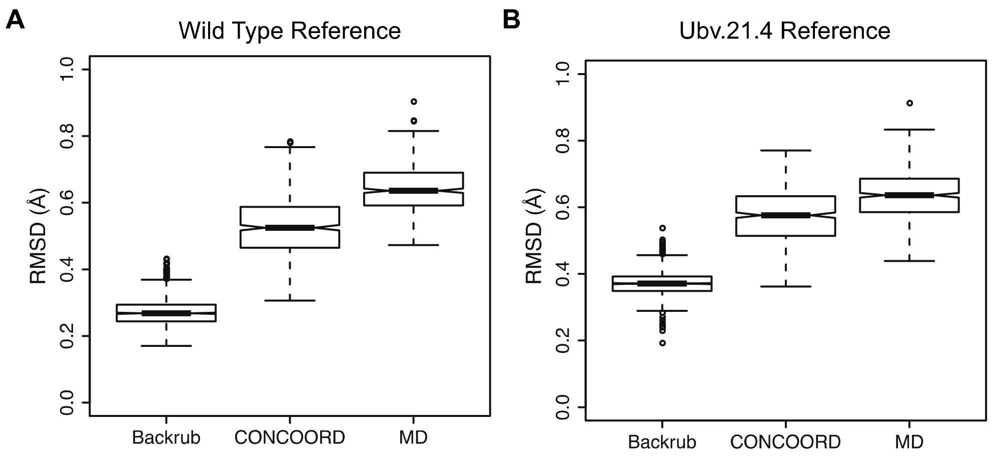

fig. S1. Structural comparisons of the ubiquitin backbone models. Computationally generated backbone models used for these comparisons are associated to the sequences selected for oligonucleotide synthesis. In total, 556, 543, and 2000 backbone models were respectively derived from MD, CONCOORD and Backrub protein design strategies. (**A**) MD, CONCOORD, and Backrub backbone models had a respective mean ubiquitin RMSD of 0.64, 0.52, and 0.27 Å with respect to a wild type ubiquitin structure in complex with USP21 (PDB id 3I3T). (**B**) MD, CONCOORD, and Backrub backbone models had a respective mean ubiquitin RMSD of 0.64, 0.57, and 0.37 Å with respect a known ubiquitin variant found to inhibit USP21 (PDB id 3MTN).

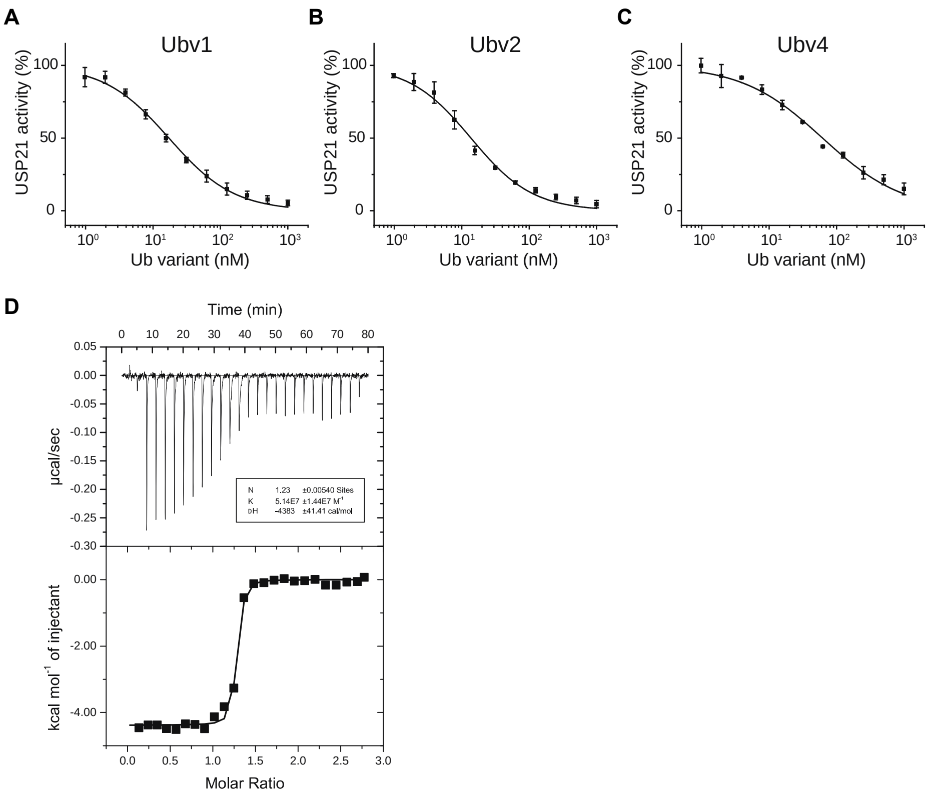

fig. S2. IC_{50} and affinity validation of a subset of the designed ubiquitin variants against USP21. (**A-C**) Dose response curves for selected designed ubiquitin variants inhibiting USP21 activity. The IC_{50} concentrations were evaluated at the ubiquitin concentrations necessary to inhibit USP21 activity by 50%. Representative dose response curves for designed ubiquitin variants whose geometric IC_{50} mean concentrations are (**A**) 13.9 nM (Ubv1), (**B**) 9.9 nM (Ubv2), (**C**) and 40.4 nM (Ubv4). (**D**) ITC validation of Ubv10, an inhibitor of USP21 with a 4.4 nM IC_{50} concentration. A representative trace measuring Ubv10 binding to USP21 is shown, whose thermodynamic properties where measured to have a K_d 42±17 nM, ΔG -10.1±0.2 Kcal·mol^{-1}, ΔH -4.8±0.3 Kcal·mol^{-1}, and -TΔS -5.4±0.6 Kcal·mol^{-1}. The values are averaged over 3 separate runs and their standard deviations shown.

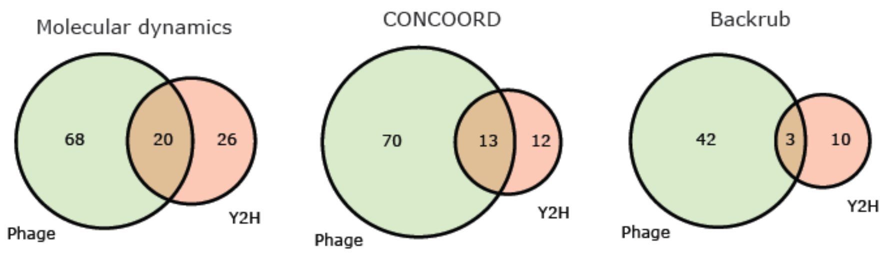

fig. S3. Venn diagrams of the designed ubiquitin variants recovered by phage display and yeast two-hybrid (Y2H).

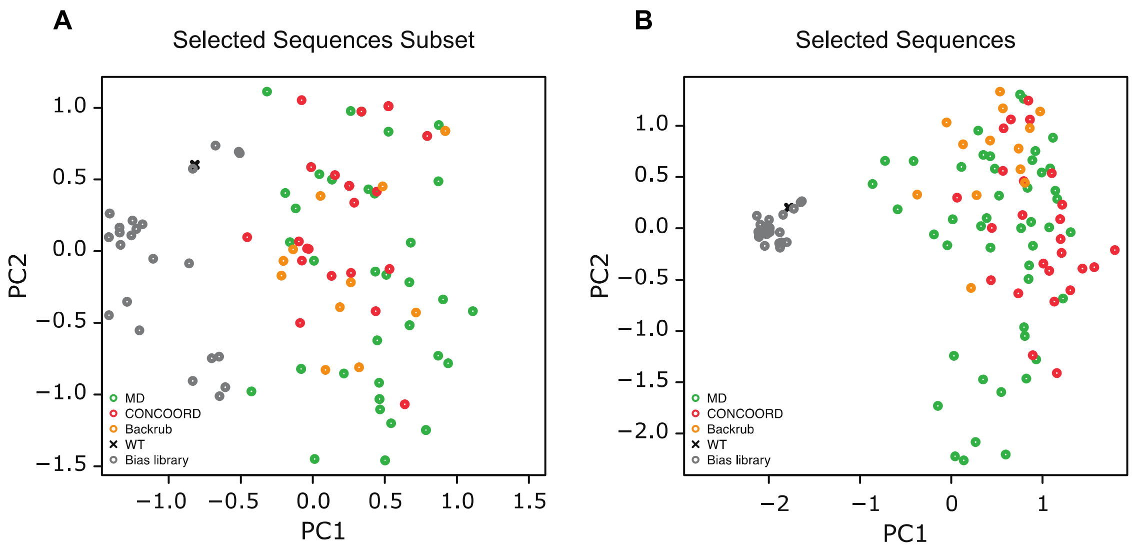

fig. S4. PCA of sequences identified by Y2H. Sequences of 84 designed variants identified by yeast two-hybrid and 26 variants from a biased naïve library found to tightly bind USP21 in addition to the wild type (WT) ubiquitin sequence were used for PCA over (**A**) 7 sequence positions (62, 63, 64, 66, 68 ,70, and 71) engineered by the biased naïve library or (**B**) over all 18 designed positions.

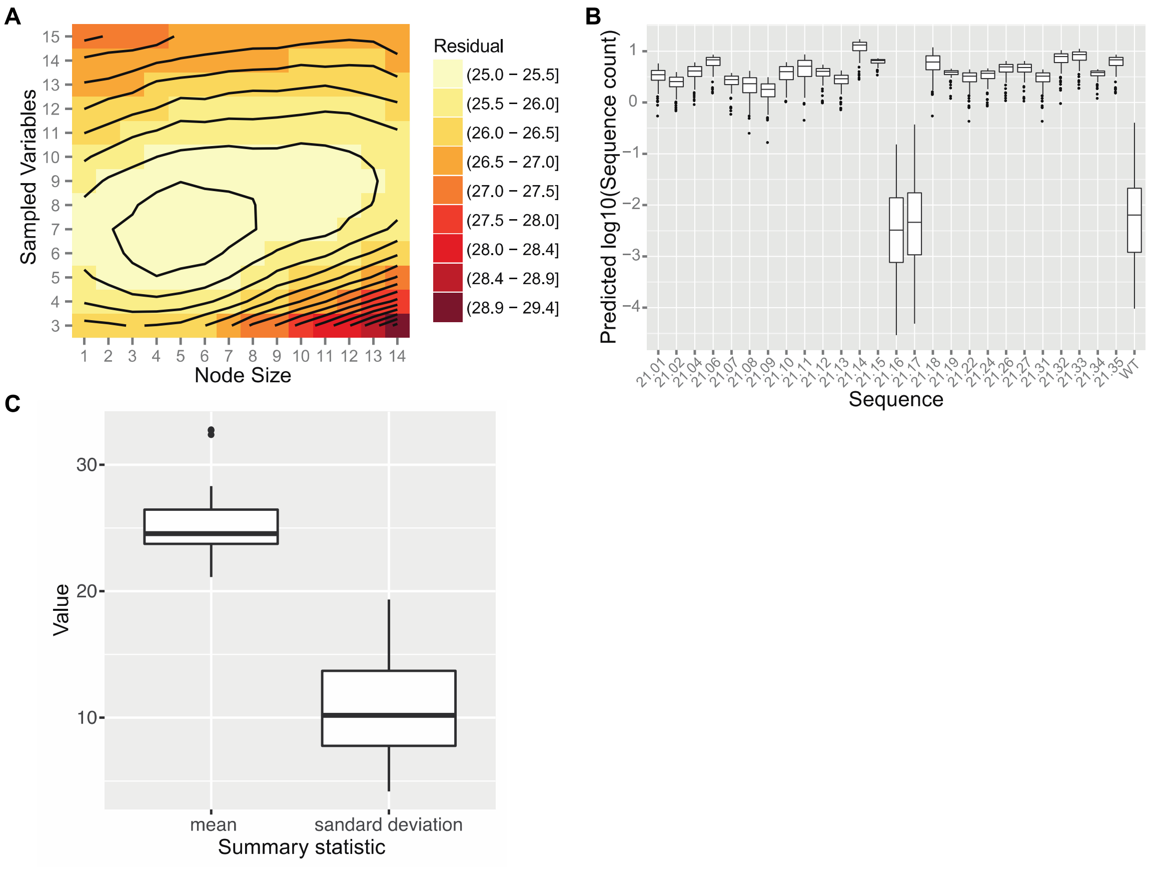

fig. S5. Random forest regression model for sequence count prediction. (**A**) Grid search over the random forest (*39*) parameters of terminal node size and the number of sampled variables chosen at each decision split. The Spearman rank correlation coefficient is used as the evaluation metric. 10 fold cross validation was performed and the average Spearman correlation coefficient was taken. The reported values were further averaged over 100 cross validation trials. (**B**) Validation of the ensemble of 100 random forest regression models on ubiquitin variants recovered from a biased naïve library (*20*). (**C**) The mean and standard deviation values after performing 5 fold cross validation with the node size and sampled variables set to 3 and 7, respectively. The values are for the 100 random forest regression models.

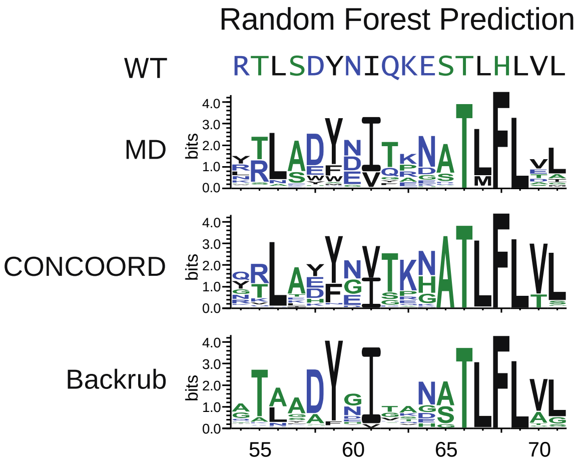

fig. S6. Sequence logos of ubiquitin variants predicted to tightly bind USP21 by an ensemble of random forests model for variants derived from MD, CONCOORD, and Backrub.

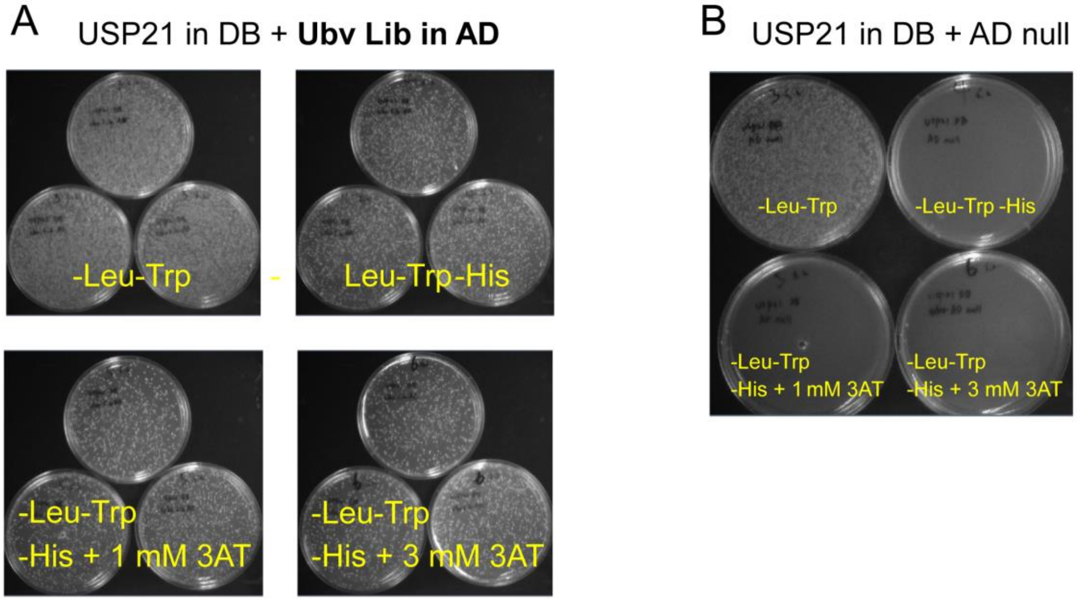

fig. S7. Y2H screening of ubiquitin library against USP21. (**A**) Diploid cells with DB-USP21 and AD-Ubiquitin library were screened on the selection plates. (**B**) Diploid DB-USP21 cells were tested for auto-activation of the GAL1::HIS3 reporter gene with pDEST-AD empty vector. No auto-activation was detected.

Supplementary Tables

table S1. Jenson-Shannon divergence of designed ubiquitin variants derived from MD, CONCOORD, and Backrub ensembles compared to the wild-type sequence and ubiquitin variants recovered from a biased naïve library (*20*). Positions not varied within the biased naïve library were set to the wild type sequence.

	Naïve lib	WT
**MD**	0.2425	0.2666
**CONCOORD**	0.3160	0.3342
**Backrub**	0.2567	0.2692
**Naïve lib**	0.0000	0.1132
**WT**	0.1132	0.0000

table S2. IC_{50} and associated deep sequencing read counts for four selected low-nanomolar binders to USP21.

	Sequence	Backbone	Read Counts	IC_{50} (nM)			
				geo mean	rep1	rep2	rep3
**Ubv1**	YPLAWYDITKFATLFLTG	MD	2210	13.9	14.1	13.7	
**Ubv2**	WTLAYYDIYRNATLFLSA	MD	9190	9.9	7.4	13.3	
**Ubv4**	YTLEYYNITKHATLFLVL	CONCOORD	98	40.4	36.6	44.5	
**Ubv10**	ATAADYDIGQNATLFLTS	Backrub	45414	4.4	4.7	4.4	4.2
**Ubv21.4**	RTLSDYNIQKWSTLFLLL			14.5	9.4	22.4	

table S3. Deep sequencing read counts ubiquitin variants surviving phage display and Y2H selections.

(See attached excel file)

table S4. Isothermal titration calorimetry of Ubv10 binding USP21.

K_a (M^{-1})	K_d (μM)	ΔG (Kcal/mol)	ΔH (Kcal/mol)	-TΔS (Kcal/mol/deg)	N	Chi^2/DoF
$2.21 \times 10^7 \pm 3.3 \times 10^6$	0.045	-10.02	-4.78	-5.24	1.15	7262
$1.76 \times 10^7 \pm 1.8 \times 10^6$	0.057	-9.88	-5.05	-4.83	1.46	9048
$4.17 \times 10^7 \pm 9.5 \times 10^6$	0.024	-10.39	-4.44	-5.96	1.24	12300
